# Review of the national usage of antibiotics in arthroplasty surgery: a need for evidence-based prescribing

**DOI:** 10.1308/rcsann.2022.0145

**Published:** 2023-06-29

**Authors:** H Hassanzadeh, A Ferro, K Woods, T Baring

**Affiliations:** Homerton University Hospital NHS Foundation Trust, UK

**Keywords:** Prophylactic, Antibiotic, Arthroplasty, Infection, Prosthetic

## Abstract

**Introduction:**

Surgical site infections (SSI) remain one of the most serious complications of arthroplasty surgery. The role of antibiotic prophylaxis in preventing SSI post-arthroplasty is well established. However, there is considerable heterogeneity in prophylactic prescribing across the United Kingdom (UK), which is contradicted by the contemporaneous evidence. This descriptive study aimed to compare the current first-line antibiotic recommendations across hospitals in the UK and The Republic of Ireland for elective arthroplasty procedures.

**Methods:**

The MicroGuide mobile phone application was used to access hospital antibiotic guidelines. First-line antibiotic recommendation and dosing regimen for primary elective arthroplasties were recorded.

**Findings:**

A total of nine distinct antibiotic regimens were identified through our search. The most frequently used first-line antibiotic was cefuroxime. This was recommended by 30 of the 83 (36.1%) hospitals in the study. This was followed by a combination of flucloxacillin and gentamicin, which was used by 38 of 124 (31%) hospitals. There was also significant heterogeneity in dosing regimens. A single prophylactic dose was most commonly recommended (52%); 4% of hospitals recommended two prophylactic doses, 19% three doses and 23% four doses.

**Conclusions:**

Single-dose prophylaxis is recognised as at least noninferior to multiple-dose prophylaxis in primary arthroplasty. There is considerable variation in the local antibiotic recommendations for surgical site prophylaxis post-primary arthroplasty surgery, with respect to both recommended first-line antibiotic and dosing regimens. With increasing emphasis on the importance of antibiotic stewardship and the emergence of antibiotic resistance, this study highlights the need for an evidence-based approach to prophylactic dosing across the UK.

## Introduction

Joint arthroplasty surgery is one of the most commonly performed and successful orthopaedic procedures in the United Kingdom (UK). Surgical site infection (SSI), and more specifically periprosthetic joint infection (PJI), remains one of the most serious complications of arthroplasty surgery.^1^ PJI presents numerous additional challenges to the usual postoperative recovery, including the need for multiple procedures, a protracted period of reduced mobility, suboptimal long-term outcomes (including the possibility of amputation of the affected limb), and higher incidence of mortality.^1,2^

From April 2018 to March 2019 a total of 44,755 elective knee replacements and 41,680 elective hip replacements were performed in England according to the national data published by Public Health England (PHE). These figures include 403 cases of revision total knee replacements and 333 cases of revision total hip replacements that were due to PJI.^3^

It is well established that antibiotic prophylaxis reduces PJI rates. A systematic review of antibiotic prophylaxis in total joint arthroplasty in 2008, encompassing 11,343 participants, identified an absolute reduction of 8%, and a relative reduction of 81%, in risk of postoperative infection with the use of antibiotic prophylaxis compared with no prophylaxis.^4^ The choice of antibiotic is necessarily not uniform between regions, and is dictated by patient characteristics (immunocompetence for example), local availability, cost and an appreciation for causative pathogens endemic to the region. The dosing regimen, however, provides greater opportunity for standardisation.

Antibiotic stewardship should be at the forefront of decisions on prophylaxis dosing regimens. The injudicious use of broad-spectrum antibiotics is associated with the establishment of multidrug-resistant pathogens,^5,6^ and this is also true in the context of PJI with the emergence of methicillin-resistant *Staphylococcus aureus* as a causative pathogen in SSI. Guidelines on surgical site prophylaxis should strive to mitigate the unnecessary use of antibiotics to achieve a balance between optimal therapeutic effect and risk of resistance. There is limited evidence to support the additional benefit of protracted courses of prophylactic antibiotics in PJI compared with single-dose prophylaxis based on observational studies.^7^ Similarly, this principle of single-dose prophylaxis is supported by high-quality evidence in postoperative infection prevention in the treatment of closed fractures.^8^

The purpose of this study was thus to compare the current first-line antibiotic recommendations across hospitals in England, Scotland, Wales, Northern Ireland and The Republic of Ireland for elective arthroplasty procedures, with a particular interest in the recommended duration of perioperative prophylaxis.

## Methods

**Figure 1:**
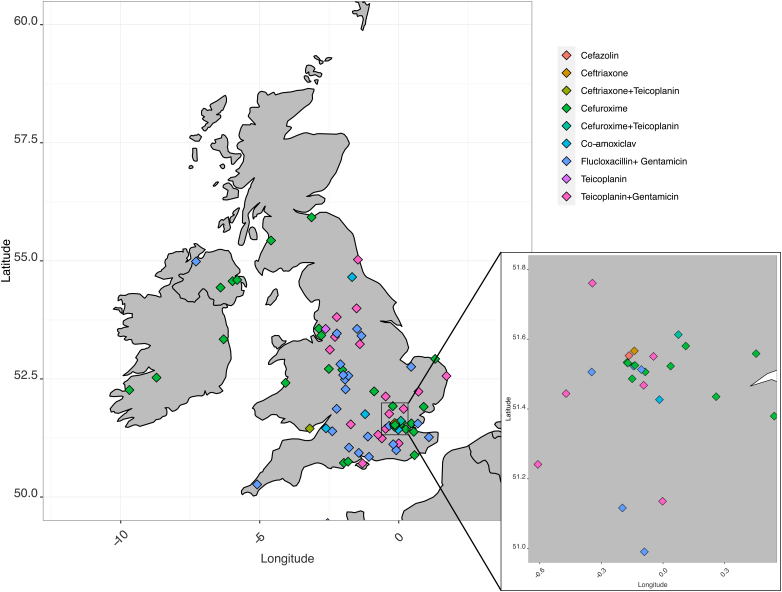
Map of included trusts and first-line antibiotics

The MicroGuide mobile phone application was used to access National Health Service trust/hospital antibiotic guidelines across the UK and Republic of Ireland. A total of 83 trusts with orthopaedic units had guidelines published for prophylactic antibiotic use in primary arthroplasty (Figure 1). Trust identities were anonymised for data extraction. Two trusts redirected to another application where they had published their guidelines and these were also included.

The recommended first-line antibiotic(s) for surgical prophylaxis for elective primary arthroplasty (upper and lower limb) in adults were recorded from each trust-published guideline. For the purpose of this study, only the first-line antibiotic choice for each trust has been compared. Data were current at the time of collection in November 2020. Figures were created using R version 3.3.2 and edited using Affinity Designer software.

## Findings

**Figure 2:**
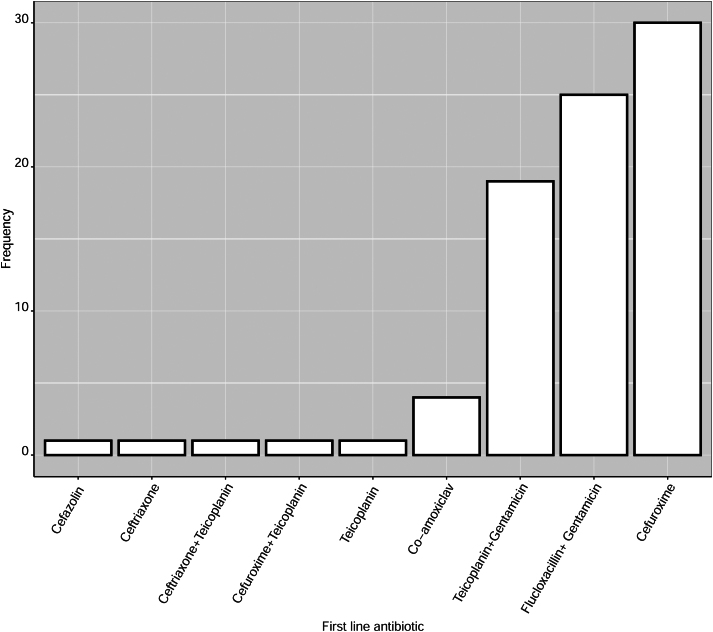
An overview of first-line antibiotic

A total of nine distinct antibiotic regimens were identified through our search. The most frequently used first-line antibiotic was cefuroxime, a second-generation cephalosporin. Cefuroxime (alone) was recommended by 30 of the 83 (36.1%) trusts in the study. This was followed by a combination of flucloxacillin and gentamicin used by 25 (30%) trusts. The third most common regimen was teicoplanin and gentamicin, used by a further 19 (23%) trusts (Figure 2). Although no clear geographical pattern for the choice of antibiotic prophylaxis was identified, it appeared that cefuroxime was the predominant choice in all countries with exception of England. The Republic of Ireland (3/3 trusts), Northern Ireland (3/4 trusts), Scotland (2/2 trusts) and Wales (2/3 trusts) recommend cefuroxime as their first choice. In England there is less consistency, and geographically proximate hospitals with overlapping patient populations often have differing prophylaxis recommendations. Two trusts were identified as having different first-line antibiotics used by each hospital within a single trust.

**Figure 3:**
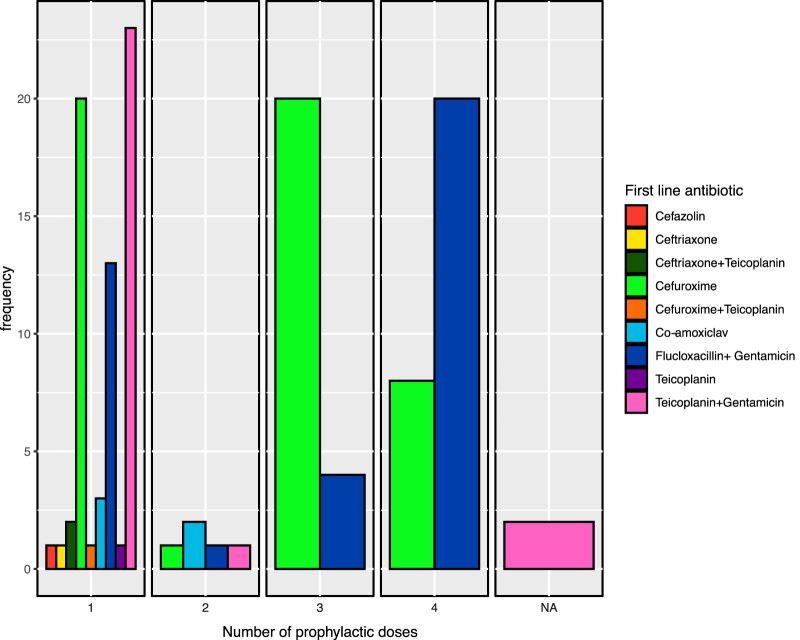
Recommended number of perioperative doses, group by first-line antibiotic

Dosing recommendations between trusts also showed significant heterogeneity, even with a single recommended antibiotic (Figure 3). The majority of antibiotics were recommended as a single perioperative dose (54%). However, 4% of trusts recommended two perioperative doses, 19% recommended three doses, and 28% recommended a total of four perioperative doses. Two trusts did not provide a specific number of prophylactic doses, and instead provided only a recommendation for the first-line antibiotic.

Of the 30 trusts recommending cefuroxime, a single stat dose was recommended by 13 trusts, two further postoperative doses recommended by 1 trust and three further postoperative doses recommended by 12 trusts. Flucloxacillin was recommended either as 1g or 2g doses. Of the 25 trusts using flucloxacillin, 18 recommended further 24-hour postoperative doses. Teicoplanin dosing recommendation was based on patient weight. Only one trust recommended a second postoperative dose of teicoplanin. Overall, 37 trusts recommended giving prophylactic antibiotics up to 24 hours postoperatively.

## Discussion

The purpose of antibiotic prophylaxis is to achieve a tissue drug level that exceeds, for the duration of the procedure, the minimum effective concentration for the pathogens likely to be encountered during the operation.^9^ As aptly put by Meehan *et al*, the goal of prophylaxis is to ‘decrease the bacterial burden at the surgical site; not to sterilize the patient’.^9^

The World Health Organization (WHO) published a global strategy in 2021 to contain antimicrobial resistance.^10^ Despite this acknowledgement, coordinated efforts have fallen short of those required to prevent the further emergence of multidrug-resistant organisms.^11^ On both a trust and clinician scale, practising sound antibiotic stewardship is fundamental in controlling this threat. This not only encompasses apportioning antibiotics in a manner that is appropriate to the causative pathogens (i.e. not prescribing unnecessarily broad-spectrum antibiotics), but also ensuring that the duration of antibiotics meets the balance between therapeutic efficacy and risk of resistance.

It is reassuring that all first-line antibiotic recommendations identified through the current study have shown efficacy against the pathogens common to postoperative SSIs in arthroplasty. However, the identified variation in first-line antibiotics is difficult to clinically justify. A comprehensive analysis of PHE’s national SSI infection database in 2016 identified that just seven organisms (at the time of study) were responsible for 89% of all infections affecting primary hip and knee arthroplasty, and that the current diversity of antibiotic regimens across England was contrary to the available evidence.^12^ The reasons for this geographic variation in antibiotic prescribing guidelines are not entirely clear. A systematic review of antibiotic usage in 2018 identified large variation in antibiotic use across similar setting and providers, and suggested several reasons for the observed variation, including prescriber preferences, resource allocation (e.g. economic incentives and organisation size), and capacity for organisation change, among others.^13^

This is also true of the recommendations for duration of prophylactic antibiotics identified through the current study. There is no strong evidence to support continuation of prophylactic antibiotics for hip and knee replacements for 24 hours postoperatively. Indeed, this is no longer recommended internationally.^14^ Observational studies support the notion that there is likely little additional risk reduction with the use of prolonged antibiotic courses when used for the purpose of prophylaxis. In 2020, Veltman *et al* conducted a retrospective observational study on risk of revision post-primary arthroplasty of the hip and knee, comparing the risk of SSI with single- vs multiple-dose prophylaxis, on a total of 242,179 patients.^7^ No significant difference was identified between the two groups. In 2017, the Centres for Disease Control and Prevention (CDC) guidelines for the prevention of SSI were updated, and recommended against the continuation of antibiotic prophylaxis (beyond that in the immediate perioperative period) in total joint arthroplasty. This recommendation applies in both clean and clean-contaminated procedures, and in the presence of a drain.^14^ Recently, a more comprehensive systematic review and meta-analysis, encompassing a total of 51,627 patients across 32 studies, was conducted to provide an updated overview of the current evidence in answering the question of duration of postoperative prophylaxis. The authors concluded that ‘the available evidence does not show added benefit of postoperative beyond 24 hours’*.* With the current body of evidence supporting single-dose prophylaxis, not only in the context of arthroplasty, but also in other orthopaedic procedures including Open Reduction and Internal Fixation (ORIF) closed long-bone fractures,^8^ the liberal use of antibiotics for durations of up to (and over) 24 hours postoperatively is increasingly difficult to justify.

The findings of the current study support that, despite the abundance of evidence supporting single-dose prophylaxis, practice continues to be varied across the UK. As clinicians invested in our patients' wellbeing, the mantle of responsibility for implementing evidence-based medicine falls squarely on our shoulders. Similarly, it is our responsibility, as those directly managing the consequences of poor antibiotic stewardship, to question current guidelines to implement change where appropriate. The authors hope that highlighting this persistence of heterogeneous antibiotic stewardship with respect to primary arthroplasty might continue to provoke discussion on this topic, with a view to the unification of current antibiotic guidelines.

A potential limitation of this paper is that not all trusts in the UK use the MicroGuide application to publish antibiotic guidelines, and so some trusts will be invariably missed.

## Conclusion

PJI is well described and there is clear evidence that antibiotics are effective in reducing the numbers of postoperative SSIs in arthroplasty cases, at least in the early perioperative phase of patient care. There is considerable heterogeneity in the antibiotics used for surgical prophylaxis for primary arthroplasty in the UK and the Republic of Ireland. Moreover, dosing regimens within one antibiotic group also vary between hospitals. This study was not designed to identify causes for this variation. There is good evidence, from both observational studies on PJI and randomised controlled trials in the context of closed fractures, that single-dose prophylaxis is at least noninferior to multiple-dose prophylactic regimes in preventing postoperative infection. It is accepted that evidence-based antibiotic regimes must be designed according to the known pathogens affecting a particular patient demographic. However, there is a good argument for the need for greater standardisation of prophylactic antibiotics in PJI across the UK, particularly in light of the pervasive risk of antibiotic resistance inherent to inappropriate antibiotic provision.
